# Knockout of p47phox inhibits deep vein thrombosis by regulating the PAC-1/FNG/MAC-1 signaling axis and ameliorating mitochondrial dysfunction

**DOI:** 10.3389/fimmu.2026.1836884

**Published:** 2026-08-12

**Authors:** YiTao Wang, Yun Wan, YiDan Zhao, XiaMin Wang

**Affiliations:** 1 Department of Orthopedics, Kunshan Hospital of Chinese Medicine, Affiliated Hospital of Yangzhou University, Kunshan, Jiangsu, China; 2 Department of Hematology, Kunshan Hospital of Chinese Medicine, Affiliated Hospital of Yangzhou University, Kunshan, Jiangsu, China; 3 Department of Critical Care Medicine, Kunshan Hospital of Chinese Medicine, Affiliated Hospital of Yangzhou University, Kunshan, Jiangsu, China

**Keywords:** deep vein thrombosis, mitochondrial dysfunction, neutrophil extracellular traps (NETs), p47phox, PAC-1/FNG/MAC-1 signaling axis

## Abstract

**Objective:**

To elucidate the role and molecular mechanism of the NADPH oxidase key subunit p47phox in deep vein thrombosis (DVT) formation by investigating its regulatory effects on neutrophil extracellular traps (NETs) generation, the PAC-1/FNG/MAC-1 signaling axis, and endothelial mitochondrial function, thereby providing a theoretical basis for targeted therapeutic strategies.

**Methods:**

Peripheral blood was collected from healthy volunteers and DVT patients to assess p47phox expression, NET-related markers, and coagulation parameters. A DVT model was established using p47phox gene knockout (p47phox^-^/^-^) mice and wild-type (WT) C57BL/6NJ mice to compare thrombus incidence, weight, and length. Platelets, neutrophils, and human umbilical vein endothelial cells (HUVECs) were isolated, and p47phox expression was experimentally manipulated. Flow cytometry was used to assess integrin αIIbβ3 activation (PAC-1 binding), MAC-1 activation levels, and ROS production. Western blot was used to analyze the expression of relevant signaling proteins and mitochondrial function proteins. Immunofluorescence was used to detect NET markers and the colocalization of PAC-1, FNG, and MAC-1. Assays for NETosis, coagulation function (prothrombin time [PT], activated partial thromboplastin time [APTT], tissue factor pathway inhibitor [TFPI], etc.), and adenosine triphosphate (ATP) production were performed for supplementary validation.

**Results:**

p47phox^-^/^-^ mice exhibited lower thrombus incidence, weight, and length compared to WT mice. DVT patients showed increased p47phox mRNA and protein expression in neutrophils. In p47phox^-^/^-^ mice, histone H3-positive signals in thrombi were reduced, along with lower levels of plasma cell-free DNA (cfDNA), myeloperoxidase (MPO), and neutrophil elastase (NE); NET release upon phorbol 12-myristate 13-acetate (PMA) stimulation was also attenuated. Platelets from p47phox^-^/^-^ mice exhibited reduced PAC-1 binding and fibrinogen (FNG) binding capacity, as well as decreased PAC-1-FNG-MAC-1 colocalization area and related signaling protein expression in thrombi. p47phox knockdown in HUVECs led to decreased ROS generation, accompanied by increased mitochondrial number, membrane potential, and ATP production.

**Conclusion:**

Systemic p47phox deficiency suppresses DVT formation by inhibiting the PAC-1/FNG/MAC-1 signaling axis, thereby affecting platelet–neutrophil interactions and NET generation, and by protecting endothelial cells through amelioration of ROS-mediated mitochondrial dysfunction. Although *in vitro* experiments suggest the involvement of these multicellular mechanisms, the specific contributions of individual cell types require further validation.

## Introduction

Venous thromboembolism (VTE) is the third leading cause of cardiovascular mortality, comprising deep vein thrombosis (DVT) and pulmonary embolism (PE). DVT is the predominant form and is a common cause of death following elective surgery and pregnancy (1). Up to half of patients with DVT may develop post-thrombotic syndrome, and approximately one-third experience recurrence within 10 years despite treatment (2). Furthermore, embolization of thrombus from DVT, which may travel to the pulmonary arteries and branches, may lead to the life-threatening complication of PE. The current clinical management of DVT primarily involves thrombolytic drugs, anticoagulants, inferior vena cava (IVC) filter placement, and thrombectomy. However, there is no unified or highly effective treatment strategy for DVT (3). Therefore, further research is needed to elucidate the formation process and influencing factors of DVT, uncover its pathological mechanisms, identify new therapeutic targets, and develop effective drugs, which are crucial for improving outcomes and reducing recurrence rates.

The pathological mechanism of DVT involves venous stasis, endothelial injury, and hypercoagulability, and the interaction between neutrophils and platelets is a central event in thrombus formation. When stimulated by various inflammatory factors such as interleukin-8 (IL-8), phorbol esters (3), or lipopolysaccharide (LPS), neutrophils release proteases and chromatin to form an extracellular fibrous DNA network known as neutrophil extracellular traps (NETs). Recent studies have shown that NETs play a key role in DVT by releasing DNA fibers, histones, and proteases that promote the coagulation cascade and platelet activation (4). First, the chromatin fiber web, a major component of NETs, attracts red blood cells in the bloodstream via its negatively charged surface, increasing the local concentration of coagulation factors, slowing blood flow, and forming a procoagulant scaffold (5, 6). Second, DNA released from NETs can activate factor XII and platelets via the receptor for advanced glycation end products (RAGE), promoting venous thrombosis (7). Furthermore, histones H3 and H4 on NETs bind to Toll-like receptor 2 (TLR2) or TLR4 on platelets, activating them to release granular contents (8, 9). Histone H4 can also activate red blood cells by causing phosphatidylserine externalization on their membrane, thereby promoting plasma fibrin formation (10). Additionally, neutrophil elastase (NE) released during NET formation can inhibit thrombus dissolution by degrading anticoagulant factors such as tissue factor pathway inhibitor (TFPI) (11) and can activate various procoagulant components (e.g., platelets and factors V, VIII, and X) to accelerate venous thrombosis (12). Finally, histones and proteases can damage vascular endothelium, which amplifies the coagulation response by releasing von Willebrand factor (vWF) and tissue factor (TF). Studies by Fuchs et al. have shown that DNase or heparin can degrade NETs and inhibit thrombus formation (13). Despite the recognized importance of NETs in venous thrombosis, their mechanisms require further investigation. Therefore, elucidating the relationship between NETs and venous thrombosis may provide new diagnostic and therapeutic avenues for the clinical management of thrombotic diseases.

Extracellular stimuli such as radiation and microbes, as well as various intracellular enzymes, can induce cells to produce reactive oxygen species (ROS), primarily via the NADPH oxidase (NOX) and mitochondrial pathways (14). Four NOX family members have been identified in vascular cells: NOX1, NOX2, NOX4, and NOX5 (15). p47phox, a key cytosolic subunit of the NOX2 complex, activates NOX2 upon phosphorylation and translocation to the membrane where it associates with other cytosolic subunits (16, 17). The phagocyte NADPH oxidase (NOX2) complex is the primary source of ROS in neutrophils, and its activation is critically dependent on the cytosolic subunit p47phox. Studies have shown that p47phox^-^/^-^ smooth muscle cells exhibit reduced superoxide production and a diminished proliferative response to growth factors. Preliminary research indicates that p47phox^-^/^-^ mice have a significantly reduced capacity for DVT formation, accompanied by decreased NETs generation within thrombi. Beyond its classical role in microbial killing, emerging evidence indicates that p47phox-derived ROS serve as key intracellular signals driving pathological neutrophil activation, including upregulation of adhesion receptors such as MAC-1 and induction of NETosis. Given that MAC-1-mediated platelet–neutrophil interactions and NETs release are both fundamental to thrombus propagation, p47phox likely constitutes a critical mechanistic hub linking neutrophil oxidative stress to prothrombotic function. However, the specific role of the p47phox–neutrophil axis in coordinating PAC-1/FNG/MAC-1 signaling and NETosis during DVT remains unclear.

Platelets express the integrin αIIbβ3 receptor on their surface. Upon platelet activation, αIIbβ3 undergoes a conformational change from a low-affinity to a high-affinity state (which can be recognized by the specific antibody PAC-1), thereby binding to plasma vWF, fibrinogen (FNG), and other molecules, leading to platelet aggregation. Activated platelets promote the release of active contents from internal dense granules and α-granules, including ADP and TXA2, which further activate platelets. This positive feedback loop recruits more platelets into the thrombus formation process. Furthermore, binding of activated integrin αIIbβ3 to its ligand FNG induces phosphorylation of platelet signaling proteins c-Src, Syk, and PLCγ2, mediating subsequent platelet responses like spreading, clot retraction, and thrombus stabilization (18). The integrin CD11b/CD18 (MAC-1) receptor is primarily expressed on neutrophils and monocytes/macrophages. Ligand binding to MAC-1 on neutrophils promotes the release of NE, induces neutrophil aggregation, damages vascular endothelial cells, and promotes fibrin deposition, thereby accelerating thrombus formation (19). Multiple ligand-receptor binding pathways exist between platelets and neutrophils. The activated integrin αIIbβ3 on platelets (the receptor recognized by PAC-1) and MAC-1 on neutrophils share a common ligand, FNG. When vascular endothelial injury or inflammation occurs, platelets and neutrophils can bind plasma FNG through their surface receptors, forming an activated αIIbβ3-FNG-MAC-1 complex (referred to in this study as the PAC-1/FNG/MAC-1 signaling axis based on its specific detection antibodies) (20). Soluble FNG within this complex is converted by thrombin into fibrin monomers, which polymerize into a network. This increases blood viscosity, entraps red blood cells, and recruits monocytes/macrophages to migrate into the subendothelial space, promoting thrombus formation.

This study demonstrates that p47phox deficiency significantly inhibits DVT formation, modifies the cellular composition of thrombi in a DVT model, and reduces NETs generation. Knockout of p47phox reduces integrin αIIbβ3 activation (as evidenced by decreased PAC-1 binding) and reduces FNG binding. Concurrently, p47phox deletion improves endothelial cell function by inhibiting ROS-dependent mitochondrial dysfunction. Therefore, p47phox mediates platelet-neutrophil interaction by regulating the PAC-1/FNG/MAC-1 signaling axis, thereby inhibiting NETs generation and thrombus formation.

## Materials and methods

### Animals

p47phox^-^/^-^ mice (21) were purchased from the Jackson Laboratory. C57BL/6NJ mice were used as controls. Genotyping of p47phox^-^/^-^ mice was performed according to the standard PCR protocol provided by the Jackson Laboratory. All experimental procedures were approved by the Ethics Committee of Kunshan Hospital of Chinese Medicine.

### DVT model

A DVT model was established using 20 C57BL/6NJ mice and 20 p47phox^-^/^-^ mice. The thrombus formation rate was calculated for each group, and six successfully modeled mice from each group were selected for subsequent experiments. Mice were anesthetized with 4% isoflurane, fixed in a supine position on a sterilized surgical platform, and the abdomen was fully exposed. The abdominal skin was disinfected with alcohol and incised along the midline to expose the abdominal cavity. The intestines were carefully externalized and covered with sterile gauze moistened with pre-warmed (37 °C) physiological saline to prevent desiccation. The IVC and its adjacent artery were bluntly dissected. Using the left renal vein as a landmark, the surrounding fascia and tissue were carefully separated. A suture was passed underneath the IVC. A thicker ligature was temporarily placed at the intended ligation site around the IVC and then removed to standardize the ligation tightness, preventing excessive or insufficient constriction. The IVC was then ligated at that site. Following ligation, the intestines were returned to the abdominal cavity, and the abdominal fascia and skin were sutured in layers. Postoperatively, mice were placed in a clean cage with sterile bedding until fully recovered from anesthesia and were allowed normal activity. Twenty-four hours after surgery, mice were euthanized. The fascia surrounding the IVC was dissected, and the vascular segment containing the thrombus was excised. Thrombus weight and length were measured and recorded photographically.

### Platelet isolation and extraction

Mouse blood (1 mL) was collected via retro-orbital bleeding from mice (8–12 weeks old) anesthetized with 4% isoflurane. Human venous blood was collected from the antecubital veins of healthy volunteers and DVT patients. Mouse blood was drawn into ACD-anticoagulated tubes, and platelets were isolated by differential centrifugation and resuspended in Tyrode’s buffer. For human platelets, a total of 5 healthy volunteers and 5 patients with confirmed DVT were included. All DVT patients were diagnosed by color Doppler ultrasonography and had not received anticoagulant or antiplatelet therapy within the past month; healthy volunteers had no history of thrombotic disease or related underlying conditions. ACD-anticoagulated venous blood was centrifuged to obtain platelet-rich plasma, which was then centrifuged, washed, and resuspended in Tyrode’s buffer. Prior to experimental use, platelets were allowed to rest at room temperature for 1 hour. All procedures involving blood collection from mice and humans were approved by the Ethics Committee of Kunshan Hospital of Chinese Medicine, Affiliated Hospital of Yangzhou University. Informed consent was obtained from all participants, and experiments involving human blood were conducted in accordance with the ethical principles of the World Medical Association (Declaration of Helsinki). All animal experiments complied with the ARRIVE guidelines and were performed according to the National Institutes of Health Guide for the Care and Use of Laboratory Animals (NIH Publication No. 8023, revised 1978).

### Immunofluorescence detection of NETs in thrombus tissue

Thrombus tissues from the mouse IVC were fixed in 4% PFA, dehydrated in 30% sucrose, embedded in OCT, and cut into 8–10 μm frozen sections. After rewarming to room temperature, sections were washed three times with PBS, permeabilized with 0.3% Triton X-100 for 15 minutes, and blocked with 10% normal goat serum plus 1% BSA for 1 hour. They were then incubated overnight at 4 °C with primary antibodies against CitH3 (ab5103, 1:400) and MPO (ab25989, 1:500). After three washes with PBS, sections were incubated with species-appropriate Alexa Fluor 488/555/647-conjugated secondary antibodies (1:800) for 1 hour at room temperature in the dark. Following three additional PBS washes, nuclei were stained with DAPI (1 μg/mL) for 5 minutes, and sections were mounted with antifade mounting medium. Images were acquired using a confocal microscope under identical parameters. NET-positive structures were defined as extracellular DAPI-positive fibrous or web-like structures that colocalized with CitH3 and MPO signals (22, 23). For each mouse, 3–5 sections were randomly selected, and at least 5 fields per section were examined. The NET-positive area or punctate structures were counted independently and blindly by two investigators.

### Immunofluorescence

Neutrophils were fixed with 4% paraformaldehyde (Biosharp Biotechnology, China, Cat# BL539A) for 30 minutes and then permeabilized with 0.1% Triton X-100 (Biosharp, China, Cat# 1805132) for 20 minutes. After washing with phosphate-buffered saline (PBS), cells were blocked with 5% bovine serum albumin (BSA) dissolved in PBS at room temperature for 2 hours. Cells were then incubated with primary antibodies overnight at 4 °C, followed by incubation with corresponding fluorescent secondary antibodies for 1 hour in the dark. Nuclei were stained with 4′,6-Diamidino-2-phenylindole for 5 minutes. Images were acquired and evaluated using a confocal microscope (OLYMPUS, Japan).

### Immunofluorescence for PAC-1/FNG/MAC-1 colocalization (thrombus sections)

To evaluate the colocalization of PAC-1, FNG, and MAC-1, dual colocalization staining was performed for PAC-1 with FNG and for FNG with MAC-1. Thrombus tissues from WT and p47phox^-^/^-^ mice were fixed in 4% paraformaldehyde, embedded in paraffin, and sectioned (5 μm). After antigen retrieval with citrate buffer (pH 6.0), sections were blocked with 5% BSA in PBS for 1 hour at room temperature. They were then incubated overnight at 4 °C with primary antibodies: anti-PAC-1 (1:200, green fluorescence) and anti-FNG (1:100, red fluorescence), or anti-FNG (1:100, green fluorescence) and anti-MAC-1 (1:150, red fluorescence). After washing, corresponding fluorescent secondary antibodies were applied for 1 hour at room temperature. Nuclei were stained with DAPI, and images were assessed using a confocal microscope (OLYMPUS).

### Electron microscopy

Platelets were fixed in 3% glutaraldehyde, dehydrated, and embedded in Epon 812. Ultrathin sections were prepared using an LKB-V ultramicrotome and then stained with uranyl acetate and lead citrate. Specimens were examined under a transmission electron microscope (JEOL 1200 EX), and images were captured using a Morada G2 digital camera.

### Platelet integrin αIIbβ3 activation level (PAC-1 binding assay)

An aliquot of 2.5 µL of washed platelets (platelet count: 2 × 10^8^/mL) was added to a flow cytometry tube containing 25 µL of Tyrode’s buffer, followed by the addition of CaCl_2_ to a final concentration of 1 mM and fluorescence-labeled antibodies. The mixture was incubated in the dark for 15 minutes. Subsequently, an activator (CRP at 0.25 µg/mL or thrombin at 0.01 U/mL) was added to stimulate the platelets for 20 minutes. The reaction was terminated by adding 200 µL of Tyrode’s buffer, and the platelets were resuspended. For mouse platelets, a fluorescence-labeled JON/A antibody was used; for human platelets, a fluorescence-labeled PAC-1 antibody was employed. The change in mean fluorescence intensity (MFI) was then detected by flow cytometry.

### Platelet binding capacity to FNG

(1) Cleaned glass slides were coated with 10 µg/mL FNG and incubated at 4 °C overnight (the coating solution was prepared using 0.1 M NaHCO^3^ solution, pH 8.3). (2) The following day, the coating solution was discarded, and the slides were washed three times with 1× PBS buffer to remove unbound FNG. (3) A 150 µL aliquot of washed platelet suspension (platelet count: 12 × 10^6^/mL) was evenly spread onto the slides and allowed to adhere in a 37 °C water bath for 90 minutes. (4) After adhesion, the liquid on the slides was discarded, and the slides were washed three times with 1× PBS buffer. Subsequently, 150 µL of pre-warmed (37 °C) 4% paraformaldehyde solution was added to fix the platelets for 15 minutes. (5) After fixation, excess paraformaldehyde solution was discarded, and the slides were washed three times with 1× PBS buffer. Then, 100 µL of 0.1% Triton X-100 (diluted in 1× PBS buffer) was added to permeabilize the cells for 1 hour. (6) After permeabilization, the slides were washed three times with 1× PBS buffer. Next, 100 µL of phalloidin (working concentration diluted in 1× PBS buffer containing 1% BSA) was added, and the slides were incubated in the dark at room temperature for 1 hour. (7) After incubation, the slides were washed three times with 1× PBS buffer, air-dried, mounted, and stored at 4 °C protected from light. (8) The platelet spreading on the slides was observed using the oil immersion mode of an inverted fluorescence microscope. Images were captured, and the spreading area of platelets in the observed field was calculated and analyzed using ImageJ software.

### Isolation of mouse peripheral blood neutrophils

Neutrophils were isolated using a gradient centrifugation method. A 15 mL centrifuge tube was carefully loaded with 3 mL of neutrophil separation medium (solarbio, China, P9201). Then, 1.5 mL of a sample diluted to 80% with the separation medium was layered on top to form a concentration gradient interface. Mouse orbital blood, anticoagulated 1:1 with sodium citrate, was mixed 1:1 with erythrocyte sedimentation medium. This mixture was carefully layered on top of the 80% separation medium without disturbing the interface. The sample was centrifuged at 800 × *g* for 20 minutes. Two white ring-shaped cell layers appeared in the plasma phase. The lower ring layer containing neutrophils was collected and mixed with washing solution. After centrifugation at 400 × *g* for 10 minutes, the supernatant was discarded. The pellet was resuspended in red blood cell lysis buffer, gently pipetted to lyse the red blood cells, and centrifuged at 300 × *g* for 10 minutes. Finally, the pellet was resuspended in cell culture medium, counted, adjusted to a standardized concentration, and the purity was verified by flow cytometry.

### MAC-1 activation level in mouse peripheral blood neutrophils

A 30 µL aliquot of neutrophils isolated from WT and p47phox^-^/^-^ mice was stimulated with 0.5 µM FNG and incubated at room temperature for 30 minutes. Subsequently, the cells were stained with PE-conjugated CD11b and FITC-conjugated Ly6G antibodies in the dark for 15 minutes. The reaction was stopped with 1× Tyrode’s Buffer. The cells were analyzed by flow cytometry. Neutrophils were gated based on Ly6G expression, and the MFI was analyzed.

### Flow cytometric gating strategy for human peripheral blood samples

Platelet gating strategy: Washed human platelets were resuspended and stained with CD61-PE and PAC-1-APC antibodies. During flow cytometry acquisition, the platelet population was first identified on the FSC-A versus SSC-A scatter plot by gating on events with low FSC and low SSC signals, which correspond to platelet size and granularity. To exclude non-platelet contaminants, CD61-positive cells were further gated on the CD61-PE versus SSC-A plot as the target platelet population. Within this gate, the fluorescence intensity of PAC-1-APC was analyzed to assess integrin αIIbβ3 activation, and the MFI was used for quantitative analysis.

Neutrophil gating strategy: Isolated human peripheral blood neutrophils were stained with CD15-FITC and MAC-1-PE antibodies. During acquisition, the granulocyte population was first identified on the FSC-A versus SSC-A plot by gating on events with high FSC and high SSC. Subsequently, CD15-positive cells were gated on the CD15-FITC versus SSC-A plot as the target neutrophil population. Within this gate, the fluorescence intensity of MAC-1-PE was analyzed, and the MFI was used for quantitative analysis.

### ROS generation detection

A total of 1.5 × 10^5^ human umbilical vein endothelial cells (HUVECs) were seeded into a 6-well plate. Cells were incubated with the DCFH-DA (Beyotime, China) working solution at a final concentration of 5 µM for 30 minutes at 37 °C. Cells were washed three times with fresh culture medium, collected, and analyzed by fluorescence-activated cell sorting.

### Mitochondrial content assay

HUVECs were seeded into confocal culture dishes and transfected with p47phox-siRNA or overexpression plasmids for 48 hours. After removal of the culture medium, the cells were incubated with pre-warmed serum-free medium containing 100 nM MitoTracker Red CMXRos probe (Beyotime) at 37 °C in a 5 % CO_2_ incubator for 30 minutes. Following incubation, the staining solution was aspirated, and the cells were washed three times with PBS to remove unbound probe. Images were acquired using a laser scanning confocal microscope (OLYMPUS) under identical exposure parameters. Quantitative analysis was performed using ImageJ software. Images were converted to 8-bit format, and a uniform threshold was applied; individual cell boundaries were then defined as regions of interest (ROIs). The MFI within each ROI was measured to reflect the relative mitochondrial content per cell. At least 30 cells per group were randomly selected for statistical analysis.

### Mitochondrial membrane potential assay

Mitochondrial membrane potential changes in HUVECs were assessed using the JC-1 fluorescent probe (Beyotime). Transfected cells were seeded in confocal culture dishes, and JC-1 working solution was prepared according to the manufacturer’s instructions. After aspirating the original culture medium, 1 mL of JC-1 staining working solution was added to each dish. Cells were incubated at 37 °C in a 5 % CO_2_ incubator for 20 minutes. After incubation, the supernatant was discarded, and cells were washed twice with JC-1 staining buffer (1×). Fresh medium was then added, and images were immediately acquired using a confocal microscope (OLYMPUS). Fluorescence images were collected in the same field of view using the red channel (excitation 525 nm, emission 590 nm, reflecting JC-1 aggregates) and the green channel (excitation 490 nm, emission 530 nm, reflecting JC-1 monomers). Using ImageJ software, the mean fluorescence intensities of red and green signals within individual cells were measured, and the red/green fluorescence intensity ratio was calculated as a relative quantitative indicator of mitochondrial membrane potential.

### Western blot

Western blot analysis was performed to detect the expression levels of relevant molecules. Tissues were lysed with radioimmunoprecipitation assay lysis buffer. The detailed procedure is as follows: sodium dodecyl sulfate-polyacrylamide gel electrophoresis (SDS-PAGE) Loading Buffer was added to the lysed neutrophil samples and, after gentle mixing, the samples were heated in a metal bath at 100 °C for 10 minutes to denature the proteins. The denatured samples were then subjected to SDS-PAGE electrophoresis with the initial voltage set at 80 V. Once the samples migrated to the interface between the stacking and separating gels, the voltage was adjusted to 120 V. Electrophoresis continued until the bromophenol blue dye front reached the bottom of the gel, after which the power was turned off, the glass plates were removed, and the separating gel was excised. Proteins were then transferred from the gel onto a polyvinylidene fluoride membrane using a wet transfer method. Following transfer, the membrane was placed in a clean container and immersed in 5 mL of blocking buffer (5% skimmed milk in Phosphate-Buffered Saline with Tween^®^ 20 [PBST]) and blocked for 1 hour at room temperature on a slow shaker. After discarding the blocking buffer, the membrane was washed with PBST and subsequently incubated overnight at 4 °C with the appropriate primary antibodies diluted in PBST containing 5% BSA. The membrane was then washed four times with PBST (10 minutes per wash) before incubation with an horseradish peroxidase (HRP)-conjugated secondary antibody for 1 hour at room temperature on a shaker. After another series of four washes with PBST (10 minutes per wash), protein bands were visualized using a chemiluminescence detection system. Specifically, the two components of an HRP chemiluminescence reagent kit were mixed in equal volumes in the dark, applied evenly onto the membrane, and the membrane was imaged using a chemiluminescence imaging system with adjusted exposure times. The grayscale intensity of protein bands was analyzed using ImageJ software. The primary antibodies used were as follows: p47phox (Santa Cruz, USA, sc-7660, 1:500); CD61 (abcam, UK, ab119992, 1:1000); CD11b (abcam, ab133357, 1:1000); CD18 (abcam, ab307406, 1:1000); Syk (abcam, ab40781, 1:1000); p-Syk (abcam, ab247353, 1:1000); Akt (abcam, ab8805, 1:1000); p-Akt (abcam, ab81283, 1:1000); PGC1α (abcam, ab176823, 1:1000); TFAM (abcam, ab307302, 1:1000); MT-CO1 (abcam, ab14705, 1:1000); CitH3 (abcam, ab5103, 1:1000); MPO (abcam, ab25989, 1:1000); β-actin (abcam, ab8226, 1:1000).

### Quantitative real-time polymerase chain reaction

Briefly, RNA was isolated from platelets and reverse-transcribed into cDNA, followed by PCR amplification using a LightCycler^®^ 480 II instrument (Roche Life Science, Switzerland). The primer sequences for p47phox were: forward, 5’-AAAGTACCGCTGTCTCGCAG-3’; reverse, 5’-CCTGGAACCCAACCATTGGA-3’. The expression level of p47phox mRNA was calculated using the 2^−ΔΔCt^ method and normalized to that of β-actin as an internal control.

### NETosis assay

Freshly isolated bone marrow neutrophils were seeded onto poly-L-lysine-coated coverslips (1 × 10^4^ cells per coverslip) and incubated at 37 °C in a 5 % CO_2_ incubator for 60 minutes. To induce NETosis, cells were stimulated with 10 nM PMA and further incubated for 4 hours under the same conditions. The reaction was terminated by the addition of ice-cold PBS. After fixation with ice-cold PBS containing 2% paraformaldehyde, cells were washed with ice-cold PBS. For specific staining, cells were incubated with Sytox Green dye and Hoechst at room temperature to label extracellular DNA and nuclei, respectively. Prior to imaging, coverslips were washed with PBS and mounted onto slides using ProLong Gold Antifade Mountant (Thermo Fisher Scientific, USA). For each coverslip, at least 5 fields of view at 20× magnification were randomly selected to estimate the number of neutrophils releasing NETs, expressed as a percentage of total neutrophils. For each experimental group, bone marrow-derived neutrophils from at least 3 independent mice were used, and the experiment was independently repeated three times.

### Plasma coagulation factor detection

Orbital blood (1 mL) from WT and p47phox^-^/^-^ mice, or venous blood from the antecubital veins of human subjects, was collected into sodium citrate anticoagulant. Samples were centrifuged at 4 °C and 3000 rpm for 5 minutes, and the supernatant was collected. The supernatant was further centrifuged at 4 °C and 12,000 × *g* for 15 minutes. The resulting plasma was used to measure the levels of coagulation factors (V, VIII, IX, XII, etc.) and TFPI, as well as to determine prothrombin time (PT) and activated partial thromboplastin time (APTT) using commercial ELISA kits (abcam, China).

### Detection of plasma cell-free DNA, myeloperoxidase, and NE

Plasma levels of cfDNA in WT mice, p47phox^-^/^-^ mice, and their corresponding DVT models were measured using the Quant-iT™ PicoGreen^®^ dsDNA Quantification Kit (Yeasen Biotechnology, China) according to the manufacturer’s instructions. The concentrations of MPO-DNA complexes and NE in plasma were detected using ELISA kits (Jianglai Biological, China). Briefly, 96-well plates were coated with 50 µL per well of MPO or NE antibody (5 µg/mL, diluted 1:500 in 0.05 mmol/L carbonate-buffered saline, pH 9.6) and incubated at 4 °C overnight. MPO-DNA complexes and NE levels were then measured following the kit protocols. Absorbance of the final reaction solutions was measured at 405 nm.

### Enzyme-linked immunosorbent assay

Plasma levels of human tumor necrosis factor-α (TNF-α) and interleukin-6 (IL-6) were measured using the Human TNF-α ELISA Kit (MBS824943, MyBiosource, USA) and the Human IL-6 ELISA Kit (MBS2021124, MyBiosource), respectively, following the manufacturers’ protocols.

### Hematoxylin and eosin staining

Twenty-four hours after DVT model establishment, thrombosed vascular segments were harvested and fixed in 4% paraformaldehyde. The tissues were then dehydrated, embedded in paraffin, and sectioned at a thickness of 5 µm. Sections were stained with H&E, mounted with resin, and observed under a light microscope to analyze the thrombus cross-sectional area in DVT model mice.

### Statistical analysis

Data are presented as the mean ± standard error of the mean (SEM). Statistical significance for comparisons between two groups was assessed using Student’s *t*-test. For comparisons involving more than two groups, significance was determined by one-way or two-way analysis of variance (ANOVA) followed by Tukey’s or Šidák’s multiple comparison tests. A *P*-value of less than 0.05 was considered statistically significant. All statistical analyses were performed using GraphPad Prism software version 10.

## Results

### p47phox deficiency inhibits DVT formation

**Figure 1:**
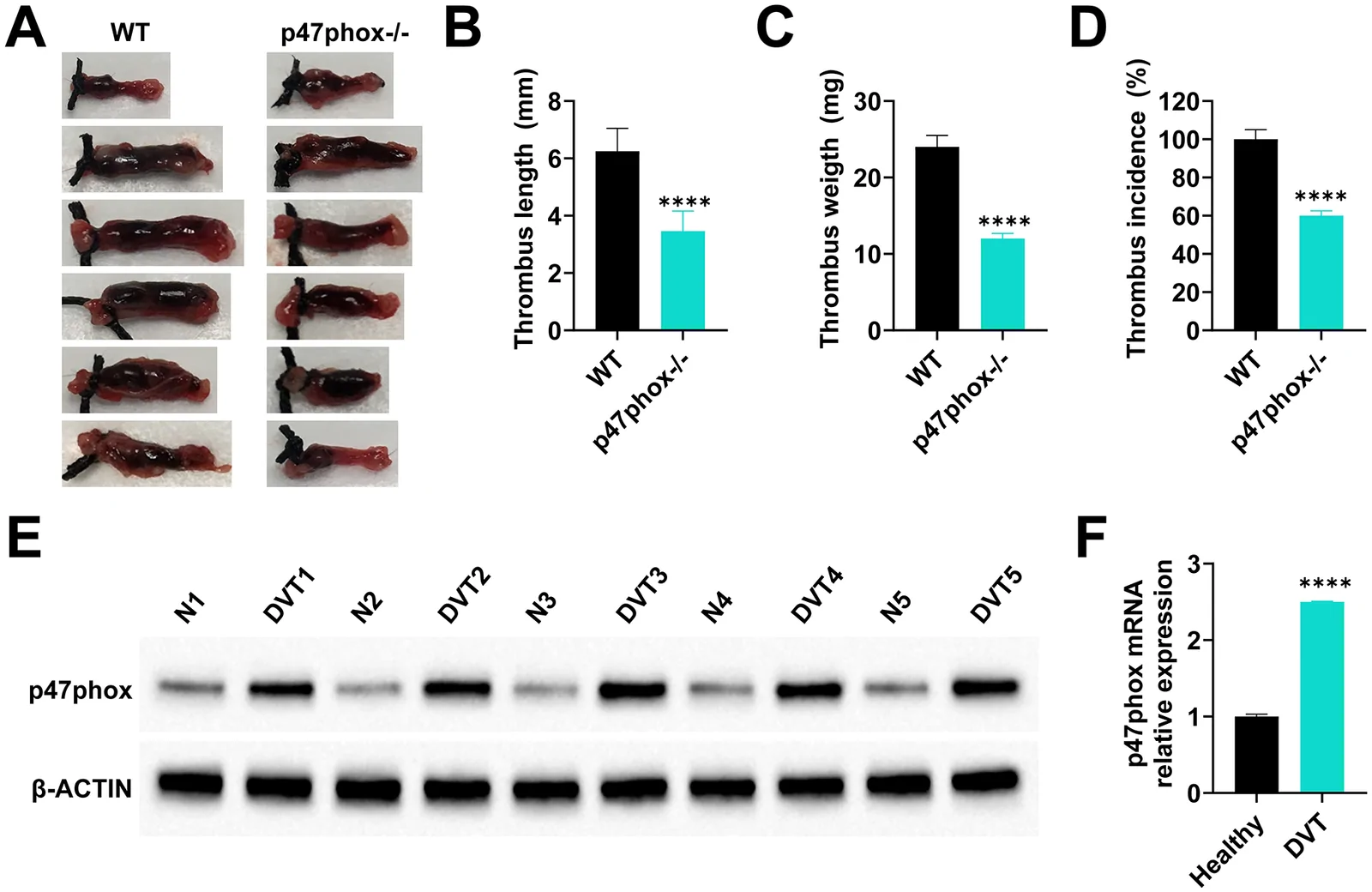
p47phox deficiency attenuates DVT formation. **(A)** Gross appearance of thrombi in WT and p47phox^-^/^-^ mice. **(B)** Thrombus length (n = 6 mice per group). **(C)** Thrombus weight (n = 6 mice per group). **(D)** Thrombus formation rate (n = 20 mice per group). **(E)** Protein expression of p47phox in neutrophils from healthy volunteers and DVT patients. **(F)** mRNA expression of p47phox in neutrophils from healthy volunteers and DVT patients. Data are presented as mean ± SD (**(B, C)** n = 6 mice per group; **(D)** n = 20 mice per group; **(E, F)** 5 healthy volunteers and 5 DVT patients). *P < 0.05, **P < 0.01, ***P < 0.001, ****P < 0.0001. Statistical significance was calculated using a two-tailed unpaired Student’s *t*-test (for two groups) or one-way ANOVA followed by Tukey’s test (for more than two groups).

A DVT model was established to simulate postoperative venous stasis. p47phox-deficient (p47phox^-^/^-^) mice exhibited a significantly reduced capacity for thrombus formation compared to wild-type (WT) mice (**Figure 1A**). Both thrombus length and weight were significantly lower in p47phox^-^/^-^ mice than in WT mice (**Figures 1B, C**). Furthermore, the thrombus incidence rate was significantly lower in p47phox^-^/^-^ mice (**Figure 1D**). In addition, p47phox expression was significantly higher in neutrophils isolated from DVT patients compared to healthy volunteers, as demonstrated by Western blot and RT-qPCR analyses (**Figures 1E, F**). Given that p47phox is also expressed in platelets and endothelial cells and regulates their functions (24), functional validation was subsequently performed using isolated cells *in vitro* to clarify the cell-specific roles of p47phox in these cell types.

### p47phox inhibits DVT formation by reducing NETs generation

**Figure 2:**
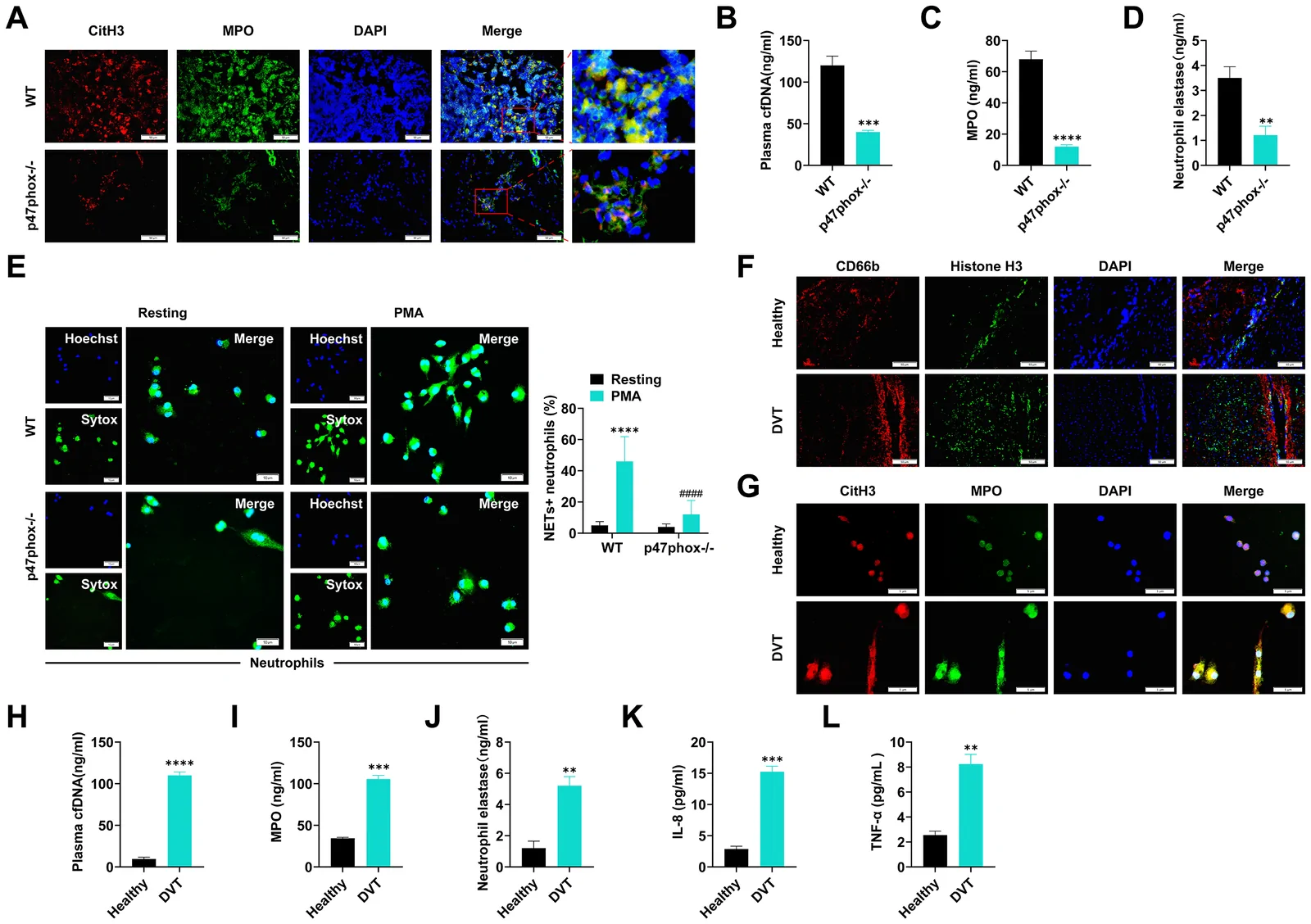
p47phox deficiency reduces NET generation. **(A)** Immunofluorescence detection of NETs in thrombi from WT and p47phox^-^/^-^ mice. Thrombus sections were co-stained with DAPI (DNA, blue), anti-CitH3 (green), and anti-MPO (red). NETs were identified as extracellular DAPI-positive filamentous or web-like structures colocalized with CitH3 and MPO signals. **(B–D)** Detection of NET-related biochemical markers in plasma from WT and p47phox^-^/^-^ mice in the DVT model group, including plasma cell-free DNA **(B)**, MPO content **(C)**, and NE **(D)**. **(E)** Sytox fluorescence staining showing reduced NETs release from p47phox^-^/^-^ neutrophils. **(F)** Immunofluorescence detection of CD66b (neutrophil marker) in neutrophils from healthy volunteers and DVT patients. **(G)** Cells were co-stained with DAPI (DNA, blue), anti-CitH3 (green), and anti-MPO (red). **(H)** Plasma cfDNA detection in healthy volunteers and DVT patients. **(I)** Plasma MPO levels in healthy volunteers and DVT patients. **(J)** Plasma NE levels in healthy volunteers and DVT patients. **(K)** Plasma IL-8 levels in healthy volunteers and DVT patients. **(L)** Plasma TNF-α levels in healthy volunteers and DVT patients. Data are presented as mean ± SD (**(A–D)** n = 6 mice per group; **(E)** bone marrow-derived neutrophils from n = 6 independent mice; **(F–L)** 5 healthy volunteers and 5 DVT patients). *P < 0.05, **P < 0.01, ***P < 0.001, ****P < 0.0001. Statistical significance was calculated using a two-tailed unpaired Student’s *t*-test (for two groups) or one-way ANOVA followed by Tukey’s test (for more than two groups).

Recent studies have shown that NETs, while participating in innate immune responses by trapping and killing pathogens, contribute to venous thrombosis through various pathways via the release of antimicrobial components such as nucleic acids, histones, and elastase (25). NETs formation is a progressive process characterized by nuclear membrane disintegration, chromatin decondensation, and cytolysis. Histone citrullination is a key marker of neutrophil chromatin decondensation, and increases in plasma cfDNA, MPO expression, and NE are also recognized as hallmarks of NETosis. Therefore, this study aimed to investigate whether p47phox deficiency reduces NETs generation. To more specifically identify NETs within thrombi, triple immunofluorescence staining for DNA, CitH3, and MPO was performed. Compared with WT mice, p47phox^-^/^-^ mice exhibited markedly fewer CitH3/MPO-double-positive web-like structures colocalized with extracellular DNA in thrombus tissues (**Figure 2A**), suggesting that p47phox deficiency suppresses intraluminal NETs formation. To further support these specific immunofluorescence findings, plasma levels of NET-related biochemical markers were measured. The results showed that plasma levels of cell-free DNA were reduced in p47phox^-^/^-^ mice compared to WT mice (**Figure 2B**), and plasma MPO and NE levels were also significantly decreased (**Figures 2C, D**). To further investigate the effect of p47phox deficiency on NETs and to determine whether this effect depends on neutrophil-intrinsic regulation, bone marrow-derived neutrophils from WT and p47phox^-^/^-^ mice were directly stimulated *in vitro* with PMA, a well-characterized NETosis inducer. Because *in vitro* stimulation eliminates interference from platelets and other microenvironmental factors *in vivo*, p47phox^-^/^-^ neutrophils exhibited significantly attenuated NET release upon PMA stimulation compared to WT neutrophils (**Figure 2E**). This result establishes a direct, cell-intrinsic regulatory role for p47phox deficiency in neutrophil NET generation. These results indicate that p47phox deficiency reduces NETs generation, thereby inhibiting DVT formation.

To confirm the identity of isolated human cells, immunofluorescence staining for the human neutrophil marker CD66b was performed. In both the healthy control and DVT groups, the vast majority of cells exhibited strong CD66b signal on the cell membrane with intact nuclear morphology, confirming successful isolation of highly enriched human neutrophils (**Figure 2F**). To more specifically identify NETs, cells were subsequently stained with DAPI, anti-citrullinated histone H3 (CitH3), and anti-MPO. In unstimulated neutrophils from healthy controls, DAPI predominantly revealed intact lobulated nuclei with minimal extracellular DNA, and CitH3/MPO signals were mainly confined to the nucleus and cytoplasm, respectively. In contrast, neutrophils from DVT patients displayed abundant DAPI-positive extracellular web-like structures colocalized with both CitH3 and MPO, indicating robust NET formation (**Figure 2G**). Concurrently, plasma levels of cell-free DNA, MPO, and NE were measured in healthy volunteers and DVT patients, and the results showed that these levels were higher in DVT patients than in healthy controls (**Figures 2H, I**). Additionally, the inflammatory cytokines IL-8 and TNF-α were measured to determine if factors such as venous stasis or endothelial injury in thrombosis stimulate NETosis. Elevated levels of both IL-8 and TNF-α were observed in DVT patients (**Figures 2K, L**), confirming the involvement of NETs in human DVT formation. These results demonstrate that p47phox deficiency reduces NETs generation, thereby inhibiting DVT formation.

### Reduced NETs generation mediated by p47phox deficiency is involved in the thrombotic coagulation process

**Figure 3:**
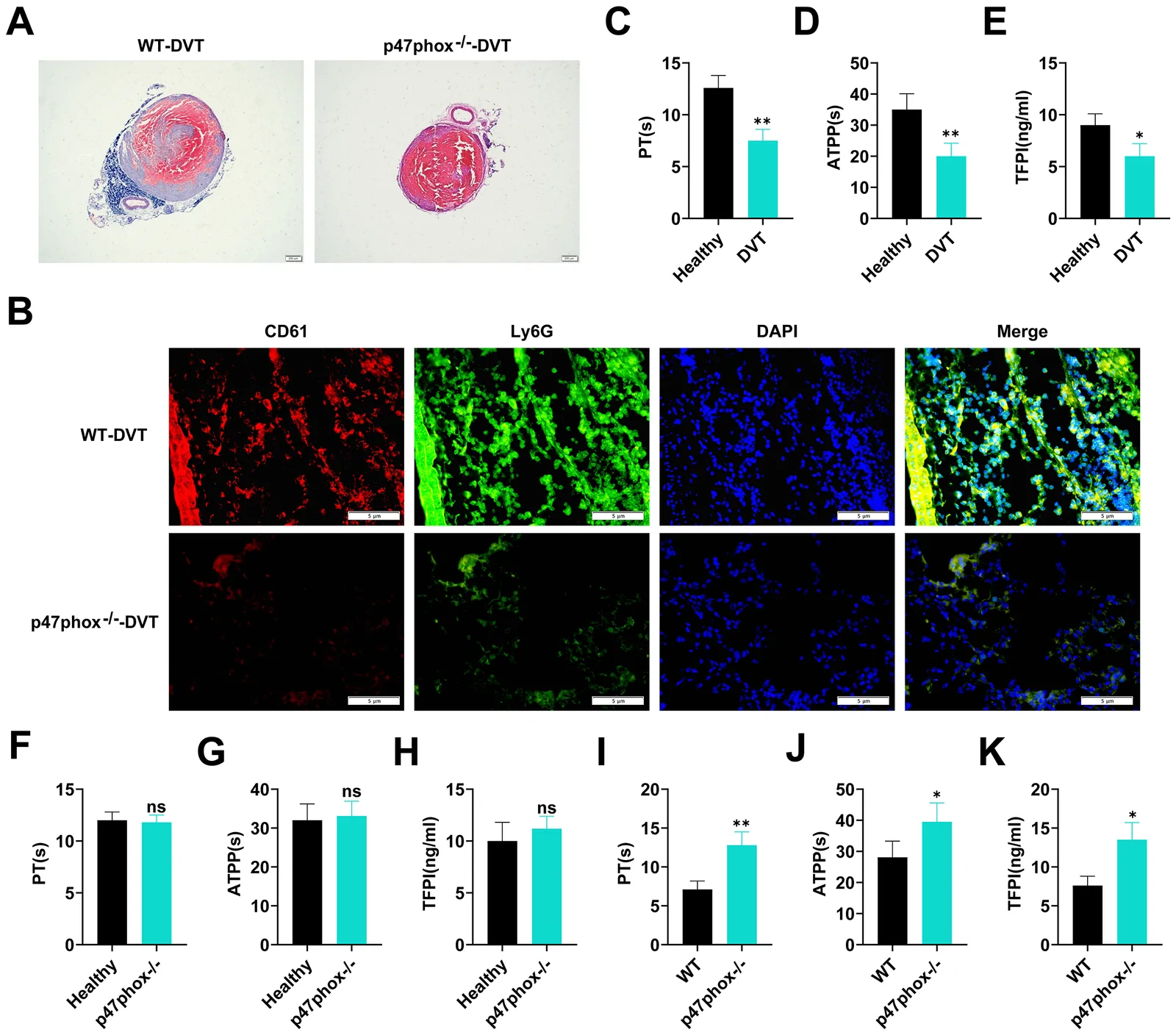
p47phox participates in the alterations of coagulation pathways in DVT via platelet-neutrophil interaction. **(A)** H&E staining showing morphological and cellular density differences in thrombi from WT and p47phox^-^/^-^ mice in the DVT model group. **(B)** Immunofluorescence staining detecting Ly6G (neutrophils) and CD61 (platelets) in thrombi from WT and p47phox^-^/^-^ mice. **(C)** PT levels in healthy volunteers and DVT patients. **(D)** APTT levels in healthy volunteers and DVT patients. **(E)** Plasma TFPI levels in healthy volunteers and DVT patients. **(F)** PT levels in plasma from naïve WT and p47phox^-^/^-^ mice. **(G)** APTT levels in plasma from naïve WT and p47phox^-^/^-^ mice. **(H)** Plasma TFPI levels in naïve WT and p47phox^-^/^-^ mice. **(I)** PT in plasma from WT and p47phox^-^/^-^ mice in the DVT model group. **(J)** APTT in plasma from WT and p47phox^-^/^-^ mice in the DVT model group. **(K)** Plasma TFPI levels in WT and p47phox^-^/^-^ mice in the DVT model group. Data are presented as mean ± SD (**(A, B)** n = 6 mice per group; **(C–E)** 5 healthy volunteers and 5 DVT patients; **(F–K)** n = 6 mice per group). *P < 0.05, **P < 0.01, ***P < 0.001, ****P < 0.0001. Statistical significance was calculated using a two-tailed unpaired Student’s *t*-test (for two groups) or one-way ANOVA followed by Tukey’s test (for more than two groups).

**Figure 4:**
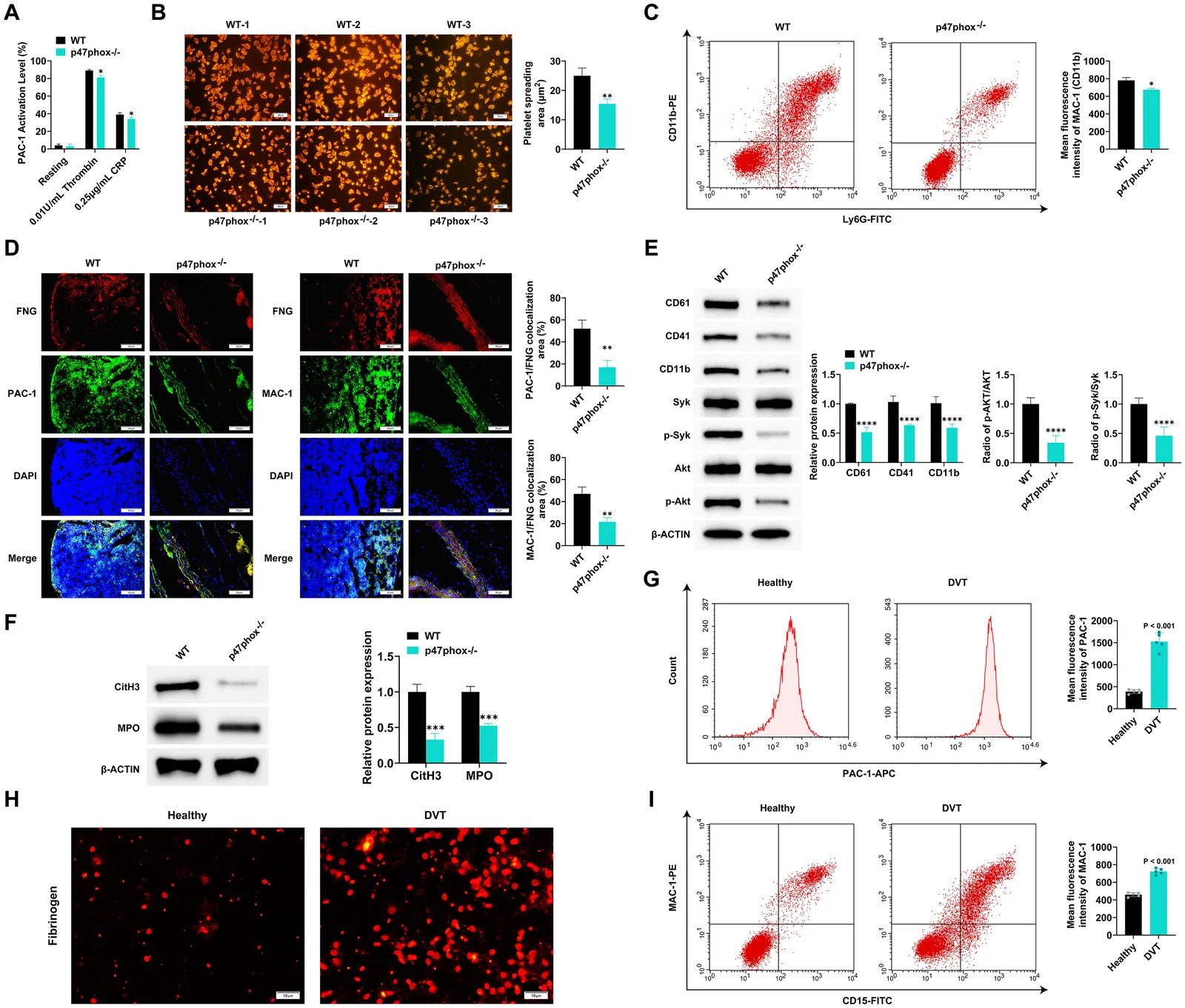
p47phox regulates the PAC-1/FNG/MAC-1 signaling axis. **(A)** Flow cytometric analysis of activated integrin αIIbβ3 (PAC-1 binding) in platelets. **(B)** Platelet spreading assay assessing the binding capacity of platelets (activated integrin αIIbβ3) to FNG from WT and p47phox^-^/^-^ mice in the DVT model group. Platelet F-actin was stained with phalloidin to visualize spreading morphology, and the coverage area was quantified using ImageJ. **(C)** Flow cytometric analysis of MAC-1 expression on the surface of neutrophils from WT and p47phox^-^/^-^ mice in the DVT model group. Gating strategy: neutrophils were gated on the FSC-A (cell size) versus SSC-A (granularity) scatter plot; Ly6G-positive cells were then confirmed on the Ly6G-FITC histogram; CD11b (MAC-1) expression was analyzed within the Ly6G-positive gate. **(D)** Colocalization analysis of PAC-1 with FNG and FNG with MAC-1 in thrombus regions. **(E)** Western blot analysis of CD61, CD11b, CD41, Syk, p-Syk, Akt, and p-Akt protein expression in thrombus tissues from WT and p47phox^-^/^-^ mice in the DVT model group. **(F)** Western blot detection of NET markers CitH3 and MPO in thrombus tissues from WT and p47phox^-^/^-^ mice in the DVT model group. **(G)** Flow cytometric analysis of PAC-1 activation level in platelets from healthy volunteers and DVT patients after activation. Gating strategy: platelets were first gated on the FSC-A/SSC-A scatter plot (low FSC/low SSC), then confirmed by CD61-PE positivity, and PAC-1-APC expression was analyzed within this gate. **(H)** Fluorescence detection of FNG binding capacity of activated platelets (activated integrin αIIbβ3) from healthy volunteers and DVT patients. Platelet F-actin was stained with phalloidin to visualize spreading morphology. **(I)** Flow cytometric analysis of MAC-1 expression in neutrophils from healthy volunteers and DVT patients. Neutrophils were gated on the FSC-A/SSC-A scatter plot (moderate FSC/high SSC), confirmed by CD15 positivity, and MAC-1-PE expression was analyzed. Data are presented as mean ± SD (**(A–F)** n = 6 mice per group; **(G–I)** 5 healthy volunteers and 5 DVT patients). *P < 0.05, **P < 0.01, ***P < 0.001, ****P < 0.0001. Statistical significance was calculated using a two-tailed unpaired Student’s *t*-test (for two groups) or one-way ANOVA followed by Tukey’s test (for more than two groups).

Studies have shown that leukocytes participate in DVT formation. Within the first 2 days of venous thrombosis, neutrophils predominate within the thrombus; from days 2 to 6, neutrophils and monocytes are the main cell types; and by day 14, monocytes become predominant (26). Preliminary research from the present group indicated that p47phox regulates hemostasis and the coagulation process in arterial and venous thrombosis by modulating platelet function. This study confirmed that p47phox deficiency reduces NETs generation, thereby inhibiting DVT formation. Therefore, whether p47phox deficiency affects platelets and neutrophils to participate in the venous thrombotic coagulation process was investigated. First, morphological changes in thrombi from WT and p47phox^-^/^-^ mice in the DVT model were examined by H&E staining. The results showed marked differences in thrombus structure between the two groups, with significantly lower cellular density in thrombi from p47phox^-^/^-^ mice compared to WT mice (**Figure 3A**). To accurately assess infiltration of specific cell populations within thrombi, immunofluorescence staining for specific markers was further performed on thrombus tissues. The results showed that the positive signal areas for the neutrophil marker Ly6G and the platelet marker CD61 were both significantly reduced in thrombi from p47phox^-^/^-^ mice compared to WT mice (**Figure 3B**). These data indicate that p47phox deficiency leads to reduced recruitment and infiltration of platelets and neutrophils in DVT thrombi. Platelets are key factors in the coagulation cascade. Therefore, whether p47phox deficiency affects the intrinsic and extrinsic coagulation pathways under physiological conditions was investigated. PT, APTT, and TFPI levels were measured in plasma from healthy volunteers and DVT patients to clarify changes in the coagulation pathways during DVT formation. The results showed that DVT patients exhibited a typical hypercoagulable state, with significantly shortened PT and APTT times and decreased TFPI levels compared to healthy volunteers (**Figures 3C–E**). This confirms the abnormal activation of systemic coagulation pathways in DVT patients. However, because human samples do not permit functional intervention of p47phox, these clinical data reflect only the disease phenotype and cannot directly elucidate the mechanism of p47phox. Therefore, the specific regulatory role of p47phox in coagulation pathways was further investigated in a mouse model. Subsequently, coagulation pathway parameters were assessed in mice. PT and APTT times were measured in plasma from naïve WT and p47phox^-^/^-^ mice. The results showed no significant difference between the two groups (**Figures 3F, G**), indicating that p47phox deficiency does not affect physiological hemostasis in normal mice. TFPI expression was also examined, revealing no change between naïve WT and p47phox^-^/^-^ mice (**Figure 3H**). This suggests that under normal physiological conditions, p47phox deficiency may not affect the intrinsic or extrinsic coagulation pathways. Subsequently, it was explored whether the reduced NETs generation caused by p47phox deficiency participates in the coagulation process under pathological venous thrombotic conditions. Accordingly, PT and APTT times were measured in plasma from WT and p47phox^-^/^-^ mice within the DVT model group. The results showed prolonged PT and APTT times in p47phox^-^/^-^ mice compared to WT mice (**Figures 3I, J**). Additionally, TFPI levels were increased in p47phox^-^/^-^ mice from the DVT model group (**Figure 3K**). These results indicate that p47phox deficiency significantly ameliorates the systemic hypercoagulable state under pathological DVT conditions. Together with the aforementioned reduction in NETs generation (**Figure 2**) and the subsequent demonstration of impaired platelet–neutrophil interactions (**Figure 4**), these findings collectively suggest that p47phox may influence coagulation pathways in DVT through regulation of platelet–neutrophil interactions and NETs generation.

### p47phox regulates the PAC-1/FNG/MAC-1 signaling axis

To investigate whether p47phox knockout affects the formation of the PAC-1–FNG–MAC-1 complex in specific cell types, flow cytometry was performed. The results showed that p47phox deficiency reduced integrin αIIbβ3 activation after platelet stimulation, as evidenced by decreased PAC-1 binding (**Figure 4A**). To verify the effect of p47phox deficiency on platelet spreading, platelet F-actin was stained with phalloidin to visualize cell morphology, and the platelet coverage area on FNG was quantified using ImageJ. The results showed that the spreading area of platelets from p47phox^-^/^-^ mice was significantly reduced compared to that of WT platelets (**Figure 4B**), suggesting that p47phox is involved in regulating platelet adhesion and spreading. To explore the colocalization of PAC-1, FNG, and MAC-1, immunofluorescence staining was performed to analyze the colocalization of PAC-1 with FNG and FNG with MAC-1 in thrombus regions. In WT mice, prominent colocalization of PAC-1 and FNG was observed in thrombus areas, suggesting platelet binding through FNG. In contrast, this colocalization was significantly reduced in p47phox^-^/^-^ mice. Similarly, the colocalization of FNG and MAC-1 was significantly higher in WT mice than in p47phox^-^/^-^ mice (**Figure 4D**), suggesting neutrophil binding through FNG. Consistent with these functional changes, Western blot analysis of thrombus lysates showed that expression levels of CD61, CD11b, CD41, p-Syk, and p-Akt were downregulated in p47phox^-^/^-^ mice compared to WT controls (**Figure 4E**). Importantly, to test whether the reduced thrombus burden was mechanistically linked to diminished NET formation, the expression of CitH3 and MPO in thrombus tissues was examined by Western blot. As shown in **Figure 4F**, the protein levels of CitH3 and MPO were significantly lower in p47phox^-^/^-^ mouse thrombi than in WT controls (**Figure 4F**). To validate the clinical relevance of p47phox in human DVT, peripheral blood samples from healthy volunteers and DVT patients were analyzed by flow cytometry. Compared to healthy controls, DVT patients exhibited significantly higher activation levels of PAC-1 on platelets (**Figure 4G**), as reflected by an increased MFI of PAC-1-APC. Concurrently, FNG binding capacity of activated platelets was also significantly enhanced in DVT patients (**Figure 4H**). Furthermore, flow cytometry was used to detect MAC-1 expression on neutrophils after FNG stimulation, and the results showed increased MAC-1 expression in DVT patients (**Figure 4I**). These findings suggest that the cellular functional changes resulting from p47phox deficiency may be involved in human DVT through the PAC-1/FNG/MAC-1 signaling axis.

### p47phox regulates endothelial mitochondrial function via ROS

**Figure 5:**
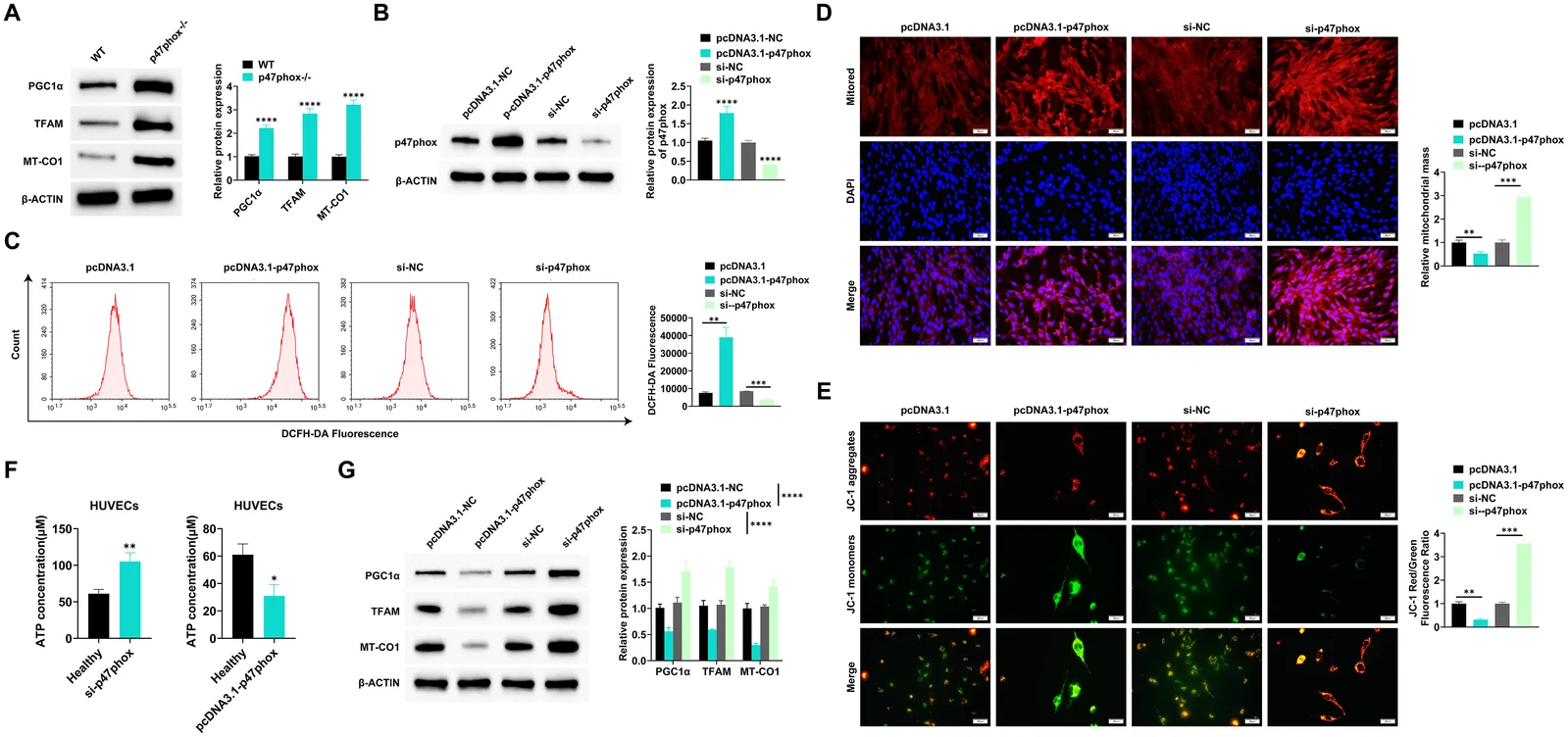
p47phox regulates endothelial mitochondrial function via ROS. **(A)** Western blot analysis of TFAM, PGC1α, and MT-CO1 protein expression in thrombus tissues from WT and p47phox^-^/^-^ mice in the DVT model group. **(B)** Detection of p47phox overexpression and knockdown efficiency in HUVECs by Western blot. **(C)** Flow cytometric analysis of DCFH-DA fluorescence intensity in HUVECs following p47phox knockdown and overexpression. Single cells were gated by FSC and SSC to exclude debris and aggregates, and DCFH-DA fluorescence intensity was analyzed within this gate. **(D)** MitoTracker Red staining detecting changes in mitochondrial mass in HUVECs following p47phox knockdown and overexpression. Left panel: representative fluorescence images; right panel: quantification of MFI per cell using ImageJ. **(E)** Measurement of mitochondrial membrane potential in HUVECs after p47phox knockdown or overexpression using the JC-1 assay. Left panel: representative fluorescence images (red: JC-1 aggregates; green: JC-1 monomers); right panel: ImageJ quantitative analysis **(F)** Western blot detection of TFAM, PGC1α, and MT-CO1 protein expression in HUVECs following p47phox knockdown and overexpression. **(G)** ATP production in HUVECs following p47phox knockdown and overexpression measured using a commercial kit. Fluorescence intensity bands were quantified using ImageJ. Data are presented as mean ± SD (**(A)** n = 6 mice per group; **(B–G)** n = 3 independent cell experiments). *P < 0.05, **P < 0.01, ***P < 0.001, ****P < 0.0001. Statistical significance was calculated using a two-tailed unpaired Student’s *t*-test (for two groups) or one-way ANOVA followed by Tukey’s test (for more than two groups).

ROS have been shown to modulate platelet function and thrombus formation (27), and it has been demonstrated that p47phox is involved in ROS production during DVT. Mitochondria are a major intracellular source of ROS, and the ROS accumulation can, in turn, impair mitochondrial function. Here, whether p47phox deficiency affects endothelial cell-specific mitochondrial function was investigated. First, mitochondrial-related proteins in thrombi from WT and p47phox^-^/^-^ mice in the DVT model were examined. The results showed increased protein expression of TFAM, PGC1α, and MT-CO1, key regulators of mitochondrial biogenesis, in the p47phox^-^/^-^ group (**Figure 5A**), suggesting that p47phox deficiency promotes mitochondrial biogenesis. To validate these findings, HUVECs, a cell line commonly used in *in vitro* studies, were selected for subsequent experiments. p47phox-siRNA and p47phox-pCDNA3.1 overexpression plasmids were transfected into HUVECs to assess cell-autonomous effects, and p47phox protein expression was confirmed (**Figure 5B**). Flow cytometry results showed that, compared to the empty vector group, knockdown of p47phox in HUVECs decreased the MFI of DCFH-DA-labeled ROS, whereas p47phox overexpression increased ROS MFI, confirming that p47phox positively regulates endothelial ROS production (**Figure 5C**). MitoTracker staining was then used to detect changes in mitochondrial mass following p47phox knockdown and overexpression. Knockdown of p47phox significantly increased MitoTracker fluorescence intensity, suggesting increased mitochondrial mass, whereas p47phox overexpression decreased mitochondrial mass (**Figure 5D**). Given that MitoTracker signal can be influenced by mitochondrial membrane potential, JC-1 staining was performed, and the results showed that p47phox knockdown increased mitochondrial membrane potential and upregulated mitochondrial biogenesis-related proteins (PGC1α, TFAM), while p47phox overexpression produced opposite effects (**Figures 5E, F**). ATP production assays further demonstrated that p47phox knockdown increased ATP production, whereas p47phox overexpression decreased ATP production (**Figure 5G**). Taken together, these results suggest that p47phox suppresses mitochondrial biogenesis and improves mitochondrial functional homeostasis in endothelial cells through regulation of ROS generation.

### Antioxidant rescue demonstrates that p47phox impairs mitochondrial function via ROS

**Figure 6:**
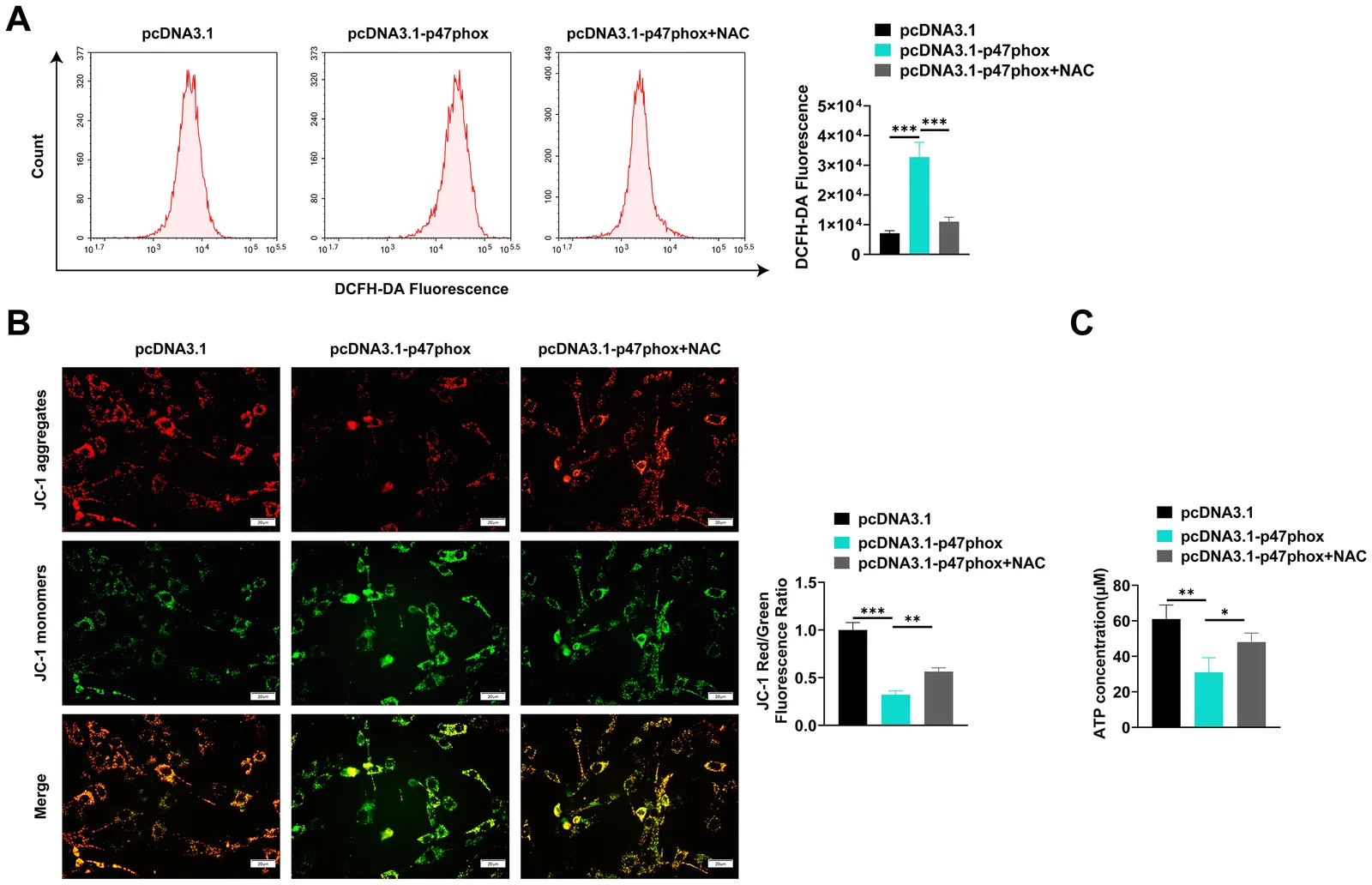
Antioxidant rescue demonstrates that p47phox impairs mitochondrial function via ROS. To scavenge intracellular ROS, N-acetylcysteine (NAC, Sigma, China) was added to the culture medium of HUVECs transfected with p47phox overexpression plasmid at a final concentration of 5 mM, and cells were incubated for 48 hours after transfection for subsequent assays. **(A)** DCFH-DA flow cytometric analysis of intracellular ROS levels. Left panel: representative histograms; right panel: quantification of MFI. **(B)** JC-1 staining for mitochondrial membrane potential. Red: JC-1 aggregates; green: JC-1 monomers; right panel: ImageJ quantitative analysis. **(C)** ATP production in HUVECs measured using a commercial kit. Data are presented as mean ± SD (n = 3 independent experiments). *P < 0.05, **P < 0.01, ***P < 0.001, ****P < 0.0001. Statistical significance was calculated using one-way ANOVA followed by Tukey’s test.

To further clarify the central mediating role of ROS in p47phox-regulated mitochondrial function, antioxidant rescue experiments were performed. In HUVECs overexpressing p47phox, the ROS scavenger N-acetylcysteine (NAC) was co-administered. Flow cytometry confirmed that NAC treatment significantly reversed ROS accumulation induced by p47phox overexpression (**Figure 6A**). More importantly, NAC effectively rescued the decrease in mitochondrial membrane potential (**Figure 6B**) and the reduction in ATP production (**Figure 6C**) caused by p47phox overexpression. These results provide direct causal evidence that p47phox impairs endothelial mitochondrial function through excessive ROS production.

## Discussion

DVT, a central pathological process of VTE, relies on the complex interplay of inflammation, coagulation, and immune responses. This study reveals for the first time that p47phox deficiency significantly reduces DVT risk by inhibiting NETs generation through regulation of the PAC-1/FNG/MAC-1 signaling axis and by improving endothelial mitochondrial dysfunction. This discovery not only deepens the understanding of the molecular mechanisms underlying DVT but also provides a novel strategy for its targeted intervention.

NETs, as an immune defense mechanism mediated by neutrophils, promote coagulation cascade activation, platelet activation, and endothelial injury under pathological conditions by releasing DNA scaffolds, histones, and proteases such as MPO and NE, ultimately driving venous thrombosis (28). The surface of NETs is decorated with various proteins and inflammatory mediators, representing a link between infection, inflammation, innate immunity, thrombosis, and cardiovascular diseases. Furthermore, NETs-associated non-neutrophil proteins, such as TF, are major triggers of the coagulation cascade and promote thrombogenesis (29). This study confirmed that in a DVT model using p47phox knockout mice, thrombus weight, length, and formation rate were significantly reduced, accompanied by decreased levels of plasma cfDNA, MPO-DNA complexes, and NE. These findings were corroborated by clinical samples, in which DVT patients exhibited elevated p47phox expression in neutrophils alongside increased NETs markers. These results directly indicate that p47phox is a key regulatory factor in NETs-driven thrombus formation. The mechanism may be related to p47phox, as a core component of the NOX2 complex, which triggers chromatin decondensation and NETs release via a burst of ROS (30, 31). This study not only demonstrates that NETs disassembly inhibits thrombosis but also further clarifies the upstream regulatory role of p47phox. Other researchers, such as Gagan D Flora et al., have also been exploring the molecular mechanisms by which NETs promote DVT. They found that the metabolic enzyme pyruvate dehydrogenase kinase (PDK) inhibits the pyruvate dehydrogenase complex, shifting pyruvate flux from oxidative phosphorylation to aerobic glycolysis. Mice with combined deficiency of PDK2 and PDK4 showed a reduced incidence of DVT, establishing a fundamental role for PDK2/4 in regulating NETosis and acute DVT (32). Chinese scholars, including Li et al., have also identified an interaction between NETs and platelet factor 4 (PF4) in DVT. Through *in vivo* and *in vitro* experiments and clinical samples, they confirmed that NETs formation is mediated by PF4 and enhances procoagulant activity in DVT (33). NETs have also been proposed as prospective biomarkers for predicting postoperative DVT, useful for estimating the probability of DVT in postoperative patients (34). Therefore, investigating the molecular mechanisms of NETs in thrombus formation in animal models and clinical contexts is of significant importance for the prevention and treatment of thrombosis and can provide new therapeutic targets.

The interaction between platelets and neutrophils is central to DVT pathology. An early study confirmed crosstalk between monocytes, neutrophils, and platelets, highlighting these interactions as unique clinical features of DVT initiation and amplification (35). Recent research by Feng et al. found that DVT is characterized by a hypercoagulable state and thromboinflammation, with significantly elevated levels of S100A8/A9 in DVT patients closely associated with platelet activation and NETs formation. The molecular mechanism primarily involves LMF as a target of S100A8/A9 affecting DVT development (36). Although triple staining was not performed in this study, colocalization of PAC-1 with FNG suggested platelet binding through FNG, while colocalization of FNG with MAC-1 suggested neutrophil binding through FNG. Taken together, these findings indicate the formation of the PAC-1/FNG/MAC-1 complex. FNG serves as a bridging molecule connecting platelets (via PAC-1) and neutrophils (via MAC-1). The higher colocalization coefficients observed in WT mice support the existence of this complex, whereas the reduced colocalization in p47phox^-^/^-^ mice indicates that p47phox regulates complex formation, consistent with the observed decrease in platelet spreading. p47phox deficiency significantly reduced integrin αIIbβ3 activation (as reflected by decreased PAC-1 binding capacity) and FNG binding capacity, and inhibited downstream phosphorylation of signaling proteins Syk, Akt, and ERK. Concurrently, the activation level of MAC-1 (CD11b/CD18) on the neutrophil surface decreased in p47phox^-^/^-^ mice, leading to weakened FNG-mediated platelet-neutrophil adhesion. In clinical samples, DVT patients exhibited enhanced platelet PAC-1 binding capacity and FNG binding capacity, along with increased neutrophil MAC-1 activation, confirming the universality of this axis in human DVT. This finding reveals that integrin αIIbβ3 activation promotes platelet aggregation and granule release. On the other hand, MAC-1 activation stimulates NE and NETs release, thereby forming a “platelet-neutrophil-coagulation” positive feedback loop. Targeting this axis may disrupt this vicious cycle, providing a precise entry point for antithrombotic therapy. Notably, in this study, p47phox deficiency affected only pathological coagulation, not physiological hemostasis. Under non-thrombotic conditions, coagulation factor activity, PT/APTT times, and TFPI levels in p47phox^-^/^-^ mice showed no difference from those in WT mice. However, in the DVT model, p47phox deficiency significantly reduced coagulation factor activity, prolonged PT/APTT, increased TFPI, and decreased neutrophil/platelet infiltration within the thrombus. This selective effect has significant clinical implications; targeting p47phox may avoid the bleeding risks associated with traditional anticoagulants, potentially achieving a therapeutic goal of “antithrombosis without anticoagulation.”

Endothelial mitochondrial dysfunction is one of the predisposing factors for thrombus formation. Xiang et al., through Mendelian randomization analysis, found no causal relationship between mitochondrial DNA copy number (mtDNA-CN) and venous thrombosis development, but suggested that VTE may lead to decreased mtDNA-CN, indicating that mtDNA-CN could serve as a biomarker for VTE in clinical practice (37). Another study found that specific mitochondrial DNA haplogroups in Native American populations increase susceptibility to venous thrombosis (38).

Furthermore, a clinical case reported an asymptomatic postpartum woman developing widespread multiple DVT, with elevated levels of M5-type mitochondrial antibodies detected (39). These studies collectively suggest a relationship between thrombus formation and mitochondrial function. In the present study, it was found that p47phox deficiency improved endothelial mitochondrial homeostasis by inhibiting ROS. In p47phox^-^/^-^ thrombus tissues, mitochondrial biogenesis markers (PGC1α, TFAM) were upregulated, suggesting enhanced mitochondrial biogenesis. *In vitro* experiments further confirmed that p47phox knockdown reduced ROS levels, increased mitochondrial mass, restored mitochondrial membrane potential, and ultimately promoted ATP production. These results indicate that p47phox-driven ROS accumulation not only suppresses mitochondrial biogenesis but also causes substantial damage to mitochondrial function (e.g., membrane potential and energy metabolism). Therefore, p47phox participates in the development of DVT through the “ROS–mitochondrial homeostasis imbalance–endothelial injury” axis. Ma et al. also discovered that ferroptosis increases the probability of DVT, primarily due to ROS generation (40). Excessive ROS can induce mitochondrial DNA damage, membrane potential collapse, and energy metabolism failure, while activation of the SIRT3/Parkin pathway can repair mitochondrial homeostasis. This study positions p47phox as a central switch in this pathway, providing a theoretical basis for antioxidant therapy in improving thrombus formation. Although the initial data showed a strong correlation between p47phox expression changes and alterations in ROS levels and mitochondrial function, correlation alone is insufficient to establish causality. To address this, NAC rescue experiments were performed, demonstrating that ROS scavenging completely reversed the mitochondrial membrane potential collapse and energy metabolism failure induced by p47phox overexpression. This critical causal evidence clearly links p47phox, ROS burst, and mitochondrial dysfunction, establishing ROS as the core effector molecule downstream of p47phox that damages mitochondria.

In addition, this study has certain limitations. A global p47phox knockout mouse model was used in this study. Since p47phox is expressed in multiple cell types including platelets, neutrophils, and endothelial cells, the global knockout model cannot completely exclude the effects of intercellular compensatory mechanisms, nor can it precisely distinguish the independent contributions of specific cell types to DVT pathogenesis. Second, a comprehensive single-cell resolution expression profile of p47phox in the thrombus microenvironment was not performed. However, the experimental design provided key complementary evidence through targeted *in vitro* and *in vivo* validation. Upregulation of p47phox expression in neutrophils from DVT patients was confirmed, and functional assays—including flow cytometry and spreading assays with isolated mouse platelets, NETosis induction in bone marrow-derived neutrophils, and p47phox knockdown/overexpression experiments in HUVECs—demonstrated the cell-autonomous regulatory effects of p47phox deficiency on these specific cell types (platelets, neutrophils, and endothelial cells), suggesting multicellular involvement in the pathological process of DVT. Nevertheless, the key cellular drivers determining the DVT phenotype in the *in vivo* microenvironment remain unclear. Future studies employing conditional cell-specific knockout mice (e.g., neutrophil-specific Lyz2-Cre or platelet-specific Pf4-Cre-driven p47phox knockout), bone marrow transplantation reconstitution, and single-cell transcriptomic sequencing are needed to more precisely dissect the decisive roles of p47phox from different cellular sources in DVT formation. Furthermore, although the genetic evidence from p47phox^-^/^-^ mice provides strong causal proof, direct translation of these findings to human cells through p47phox knockdown remains challenging because primary neutrophils are terminally differentiated and resistant to transfection. Future studies using specific pharmacological inhibitors of p47phox or optimized gene-silencing techniques in human neutrophils are needed to further validate p47phox as a viable therapeutic target for preventing DVT in clinical practice.

## Conclusion

This study demonstrates that p47phox deficiency significantly reduces DVT risk by inhibiting NETs generation through suppression of the PAC-1/FNG/MAC-1 signaling axis and by alleviating endothelial injury through promotion of mitochondrial biogenesis and improvement of mitochondrial functional homeostasis. *In vitro* cell experiments further confirmed the direct regulatory effects of p47phox on platelets, neutrophils, and endothelial cells. Targeting p47phox may provide a novel strategy for the clinical treatment of venous thrombosis; however, the specific expression profiles and driving mechanisms of p47phox in different cell populations require further elucidation using higher-resolution cellular techniques.

## Data Availability

The raw data supporting the conclusions of this article will be made available by the authors, without undue reservation.
