# Supplementary material for: Knockout of p47phox inhibits deep vein thrombosis by regulating the PAC-1/FNG/MAC-1 signaling axis and ameliorating mitochondrial dysfunction
Source: Front Immunol. 2026 Aug 12;17:1836884. doi: 10.3389/fimmu.2026.1836884 (PMC13506886; doi:10.3389/fimmu.2026.1836884)
Supplement: Supplementary file 1 [file SupplementaryFile1.pdf]

frontiers

The International Society for the Advancement of Cytometry (ISAC) has highlighted the importance of including comprehensive methodological information to ensure data reproducibility and reliability.In line with this,Frontiers in Immunology now requires authors to submit a checklist for manuscripts that involve flow or mass cytometry.This checklist helps standardize the reporting process,improving the quality and transparency of published data.By doing so,we support scientific progress,making it easier for other researchers to replicate and validate experiments.

This form should be submitted with any manuscripts using flow or mass cytometry.

Sample/specimen/material description

☑ Total blood

___PBMCs

organ digests

Other isolated platelets,neutrophils,HUVECs

Did the samples suffer any treatment before or after incubation with the antibodies?

☑ ____ Cell permeabilization...............

☑ ___ Propidium iodine

___ Not applicable

Other_

Instrument and antibodies

DB FACSCanto II

Antibodies and targets	Fluorochrome/Metal	Catalog number/Company
**anti-CD61**	**PE**	BioLegend 303906
**anti-PAC-1**	**APC**	BD 340544
**anti-CD11b**	**PE**	BioLegend 101206
**anti-Ly6G**	**FITC**	BioLegend 127602
**anti-CD15**	**FITC**	BioLegend 323006

Data analyses

1. Name of the software GraphPad Prism 10,ImageJ

2.Reference gating strategy in the manuscript or supplementary material Gating strategy in (eg Figure X) Figure 4C, 4G, and 4I legends
